# Robotic, Laparoscopic, and Open Segmental Resection vs. Extended Colectomy for Transverse Colon and Splenic Flexure Cancers: A Systematic Review

**DOI:** 10.7759/cureus.100505

**Published:** 2025-12-31

**Authors:** Sathyaseelan Arumugam, Shaurya Aggarwal, Anush Nagotu, Gary Atkin, Vivek Gupta

**Affiliations:** 1 General and Colorectal Surgery, Lister Hospital, East and North Hertfordshire Teaching NHS Trust, Stevenage, GBR

**Keywords:** colectomy, extended colectomy, minimally invasive colectomy, robotic colectomy, segmental colonic resection, splenic flexure cancer, transverse colon cancer

## Abstract

Colorectal cancer is among the most common cancers in the world. Transverse colon cancer (TCC) and splenic flexure colon cancer (SFCC) are less common entities and present distinct clinical and surgical challenges. There is still no consensus on the surgical treatment of TCC and SFCC, and the optimal extent of resection and lymph node dissection remains controversial. This study aims to evaluate whether segmental resections, performed via robotic, laparoscopic, or open approaches, provide perioperative safety and long-term oncologic outcomes comparable to those of extended colectomies in patients with TCC or SFCC. This systematic review adhered to the Preferred Reporting Items for Systematic Reviews and Meta-Analyses (PRISMA) guidelines. Relevant articles were sought in the databases PubMed, the Cochrane Library, Embase, Google Scholar, Scopus, ScienceDirect, and Web of Science, using manual search queries, up to June 30, 2024. The seven included studies comprised 454 TCCs (78 robotic, 305 laparoscopic, 71 open) and 143 SFCC, which were directly comparable by approach (39 robotic, 104 laparoscopic). Two large datasets contributed a further 10,461 SFCC cases for extent-of-resection analysis (5,698 segmental; 4,763 extended resections). Extent was reported in 454 TCC cases, with 390 segmental and 64 extended resections. Mortality was <1% across all series with no technique-related differences. Minimally invasive robotic and laparoscopic approaches have comparable safety profiles, similar minor and major complication rates, equivalent R0 resection rates, and similar anastomotic leak rates. Robotic surgery offers slightly shorter hospital stays. Both techniques remain effective and feasible for colorectal cancer surgeries, with the robotic approach providing perioperative advantages in selected cases. Current evidence supports the safety and oncologic adequacy of both segmental and extended resections. Although robotic surgery may provide modest perioperative advantages, selection of the surgical approach should be individualized, with careful consideration of surgeon experience, cost implications, institutional capabilities, and tumor-specific anatomical factors to optimize patient outcomes.

## Introduction and background

Transverse colon cancer constitutes approximately 10% of all colorectal malignancies [1]. It is often diagnosed at a later stage, which contributes to more advanced disease at the time of detection [2]. Although its overall incidence is relatively low, transverse colon cancer poses significant clinical challenges owing to its anatomical location, variable lymphatic drainage, and proximity to major vascular structures. Reported five-year survival rates range from 28% to 50%, which are lower than those for many other types of colorectal cancer [3]. Cancers arising at the splenic flexure are even less common, representing only 2-8% of colorectal cancers, with true incidence likely closer to the lower end of this range [4]. These tumors typically occur at the junction of the transverse and descending colon near the spleen. Due to their nonspecific symptoms and difficult presentation, splenic flexure cancers are frequently diagnosed at an advanced stage. The anatomical features of the transverse colon also increase the risk of adjacent organ invasion, making surgical resection particularly demanding.

Both transverse colon and splenic flexure cancers arise in anatomical "watershed" regions with variable vascular supply, which can complicate surgical planning and influence the extent of resection. This shared characteristic underlies the clinical interest in comparing segmental versus extended colectomies for these tumors and justifies evaluating both conditions together in a single systematic review. The surgical management of both transverse colon and splenic flexure cancers generally follows similar principles. Extended right hemicolectomy (ERC) remains the preferred procedure for tumors near the hepatic flexure or those involving large portions of the transverse colon [5]. This technique includes ligation of the ileocolic, right colic, and middle colic vessels, along with an extensive lymph node dissection, providing adequate oncologic clearance. Segmental resections generally involve less extensive bowel mobilization and do not require ligation of major vascular pedicles, in contrast to extended colectomies. These technical differences may influence operative complexity, perioperative outcomes, and recovery, while still aiming to achieve oncologic adequacy. For splenic flexure cancers, ERC is commonly performed as well, alongside alternative procedures such as extended left hemicolectomy or segmental splenic flexure resection [6].

In recent years, minimally invasive surgery, including both laparoscopic and robotic techniques, has been increasingly adopted for colorectal cancers. These approaches offer several advantages, including reduced intraoperative blood loss, less postoperative pain, faster recovery, and shorter hospital stays, while maintaining oncologic outcomes comparable to those of open surgery. With continued advances, minimally invasive robotic and laparoscopic segmental resection and extended colectomies (EC) have emerged as widely practiced techniques.

This systematic review aims to evaluate whether, in patients with transverse colon or splenic flexure cancers (Population), segmental resection performed using robotic, laparoscopic, or open approaches (Intervention), compared with extended colectomies (Comparison), achieves comparable perioperative safety and long-term oncologic outcomes (Outcomes). The review seeks to determine whether less extensive, minimally invasive resections yield oncologic and clinical outcomes equivalent to those of more extensive colectomies.

## Review

Methods

Study Design and Ethical Considerations

This review was conducted in accordance with the Preferred Reporting Items for Systematic Reviews and Meta-Analyses (PRISMA) guidelines [7]. Since only previously published studies were analyzed without any modifications to original patient data, ethics committee approval or individual informed consent was not required.

Protocol Registration

This systematic review was not prospectively registered in a public database such as PROSPERO. The review methodology was developed a priori in accordance with PRISMA guidelines, and all eligibility criteria, outcomes of interest, and analytical methods were defined before study selection commenced. The absence of protocol registration is acknowledged as a limitation.

Search Methodology

A comprehensive literature search was conducted in the following electronic databases: PubMed/MEDLINE, Embase, the Cochrane Library, Scopus, Web of Science, ScienceDirect, and Google Scholar. These databases were selected to ensure broad coverage of biomedical, surgical, and multidisciplinary literature, including peer-reviewed journals and conference-indexed studies relevant to colorectal surgery. The search covered all records published up to June 30, 2024, and was restricted to English-language studies involving human subjects. No lower date limit was applied.

The search strategy combined controlled vocabulary (MeSH/Emtree terms where applicable) and free-text keywords. The core search string used in PubMed was ("transverse colon cancer" OR "splenic flexure cancer") AND ("segmental colonic resection" or "colectomy" or "extended colectomy") AND ("robotic colectomy" or "minimally invasive colectomy"). This strategy was adapted to account for syntactic and indexing differences across databases.

To identify additional eligible studies, a snowballing technique was employed by manually reviewing the reference lists of all included articles and relevant review papers. Additionally, PubMed's "related articles" function was used to identify potentially relevant studies not captured by the initial search.

Inclusion and Exclusion Criteria

We selected articles published in English that reported patients undergoing robotic, laparoscopic, and open segmental and extended colectomy for splenic flexure and transverse colon cancer. Studies were excluded if they were case reports, abstracts, posters, letters to the editor, study protocols, non-human studies, editorials, clinical cases, animal experiments, or lacked sufficient or relevant clinical data.

Study Selection

**Figure 1:**
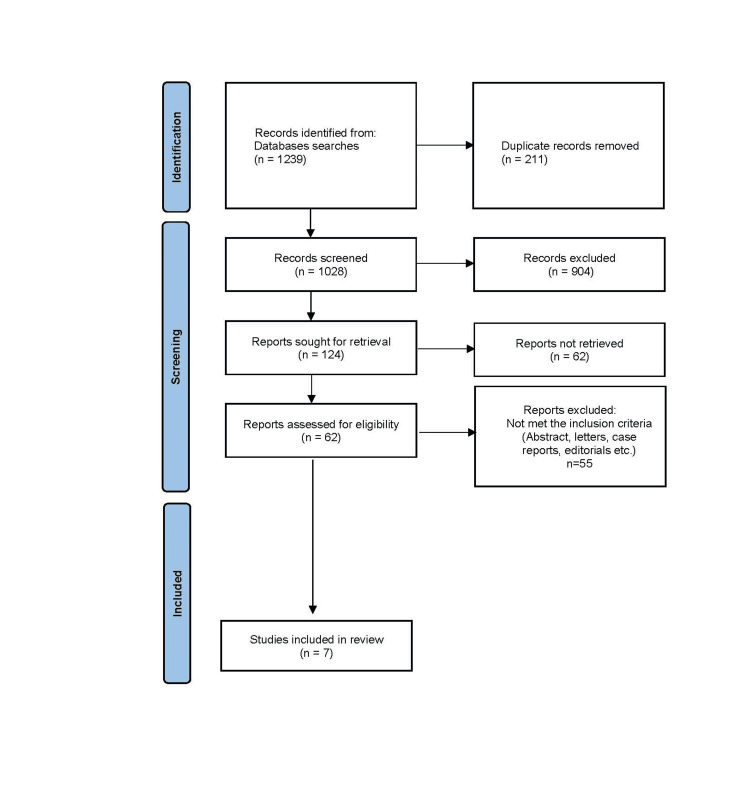
PRISMA flowchart depicting the study selection. PRISMA: Preferred Reporting Items for Systematic Reviews and Meta-Analyses

The selection process consisted of two phases. First, Shaurya Aggarwal and Anush Nagotu independently screened the titles and abstracts of all identified studies. The third reviewer, Sathyaseelan Arumugam, resolved any disagreements to make the final inclusion decision. Studies that did not meet the inclusion criteria were excluded. In the second phase, the reviewers re-evaluated the studies by thoroughly reading the full texts. The search results, as well as the process of screening and selection of studies, are presented in the PRISMA flow diagram (Figure 1). Any discrepancies in extracted data were resolved through discussion among the reviewers, with final consensus achieved in all cases.

Data Synthesis and Analysis

Given the substantial heterogeneity among the included studies in terms of study design, patient populations, surgical approaches, extent of resection, and reported outcomes, a quantitative meta-analysis was not performed. Instead, a structured narrative synthesis was conducted in accordance with guidance from the Cochrane Handbook for Systematic Reviews of Interventions, particularly for situations in which statistical pooling is inappropriate.

Studies were grouped according to tumor location (transverse colon vs. splenic flexure), surgical approach (robotic, laparoscopic, or open), and extent of resection (segmental vs. extended colectomy). Within each group, outcomes were synthesized descriptively across four domains: perioperative outcomes (mortality, morbidity, length of stay), technical outcomes (operative time, blood loss), oncologic adequacy (lymph node harvest, R0 resection), and survival outcomes, where reported.

When findings were consistent across studies, conclusions were drawn based on the convergence of results. In cases of conflicting findings, greater weight was given to larger sample sizes, propensity-matched analyses, and population-based datasets. A conclusion of "no significant difference" was reported only when multiple studies demonstrated comparable outcomes without consistent directional trends favoring one approach. Individual study statistics (p-values and confidence intervals) are reported descriptively to support observed trends rather than to imply pooled statistical inference.

Study Characteristics

A total of 1,239 records were identified from database searches (Figure 1), and after removing 211 duplicates, 1,028 unique records were screened. Of these, 904 records were excluded based on initial screening criteria. Full-text reports were sought for 124 studies, but 62 could not be retrieved despite exhaustive retrieval attempts, including institutional access, interlibrary loans, and author contact. Among the 62 reports assessed for eligibility, 55 were excluded for not meeting the inclusion criteria, and seven studies were included. Among the seven studies, four studies purely compared the outcomes of the robotic vs. laparoscopic approach. One study compared the outcomes of robotic, laparoscopic, and open approaches. The remaining two studies compared the extent of resection analysis. Across the seven included studies, a total of 454 transverse colon cancer cases were identified, comprising 78 robotic, 305 laparoscopic, and 71 open colectomies. For splenic flexure cancers, 143 minimally invasive cases were directly comparable by surgical approach (39 robotic and 104 laparoscopic). In addition, two large database studies contributed a further 10,461 splenic flexure cancer resections for extent-of-resection analysis, consisting of 5,698 segmental colectomies and 4,763 extended colectomies. Among the primary transverse colon cancer studies, the extent of resection was reported in 454 patients: 390 underwent segmental colectomy, and 64 underwent extended colectomy. Table 1 summarizes the characteristics of the included studies.

**Table 1:** Characteristics of the included studies.

Study (Authors, Year)	Study Design	Data Source/Setting	Number of Patients	Population	Surgical Intervention(s)	Outcomes Assessed	Key Findings
de’Angelis N et al., 2015 [8]	Retrospective matched case-control	Two surgical centers (France & Belgium); patients with colon cancer requiring colectomy	44 (Laparoscopic: 22, Robotic: 22)	Transverse colon cancer patients	Transverse colectomy (segmental)	Operative time, blood loss, complications, conversions, mortality, length of stay, lymph node harvest, surgeon's ergonomics	• Similar oncologic adequacy (Lymph node harvest, margins) • Longer operative time with robotics • Comparable postoperative complications
Milone et al., 2022 [9]	Multi-center observational cohort with propensity score matching	Multi-center (Italy)	164 (Laparoscopic: 146, Robotic: 18)	Transverse colon cancer patients	Transverse colectomy (segmental)	Operative variables, postoperative morbidity, oncologic adequacy	• MIS associated with shorter LOS, fewer complications • No difference in oncologic outcomes
Maertens et al., 2022 [10]	Retrospective cohort	Single tertiary center, prospectively maintained data	246 (Laparoscopic: 137, Robotic: 38, Open: 71)	Transverse colon cancer patients	Not specified, determined by the surgeon	Perioperative complications, functional and oncologic results	• Robotic & lap had fewer complications and shorter LOS than open • No significant differences in survival or oncologic results
Pang AJ et al., 2022 [11]	Retrospective database study	ACS-NSQIP colectomy data	3049 (Segmental colectomy: 2550, left hemicolectomy: 499)	Elective splenic flexure colon cancer patients	Segmental colectomy vs. left hemicolectomy	Lymph node harvest, operative time, morbidity	• Adequate lymph node harvest • Acceptable morbidity rates • Safe operative profile in large dataset
Kim et al., 2018 [12]	Retrospective cohort	Single-center, elective colorectal resections	73 (Laparoscopic: 53, Robotic: 23)	Splenic flexure adenocarcinoma patients	Left colectomy	mortality, length of stay, lymph node harvest, disease-free survival, ileus incidence, anastomotic leak	• Robotic: lower blood loss, better mesocolic excision quality • Similar complications & LOS • Operative time longer in robotic
Zhang T et al., 2021 [13]	Propensity score matched cohort	Single-center hospital	70 (Laparoscopic: 51, Robotic: 19)	Splenic flexure colon cancer patients	Segmental splenic flexure colectomy	Operative safety, lymph nodes, complications	• Safe and feasible with low complication rates • Adequate LN harvest and margins • Short-term outcomes comparable or slightly improved vs. lap
Kohn J et al., 2024 [14]	Retrospective observational study	Single-center	7412 (Segmental colectomy: 3148, Extended colectomy: 4264)	Splenic flexure adenocarcinoma patients	Segmental colectomy vs. extended colectomy	Perioperative morbidity, oncologic outcomes	• Less extensive resections (segmental) did not compromise oncologic outcomes • Lower morbidity with limited resections

A meta-analysis was not performed in this review due to significant heterogeneity among the included studies in terms of study design, surgical approaches, and reported outcomes. This variability limited the feasibility of quantitatively combining the data, thereby justifying the use of a narrative synthesis approach to summarize and interpret the findings.

Results

The seven included studies, primarily retrospective cohorts, offer low-certainty evidence comparing surgical approaches and the extent of resection for transverse colon and splenic flexure cancers. Across these studies, no significant differences were observed in mortality, major morbidity, anastomotic leak rates, or severe complications when comparing robotic, laparoscopic, and open techniques, or segmental versus extended colectomies. Perioperative outcomes, including hospital stay and reoperation rates, were also comparable between the approaches. Similarly, oncologic outcomes, including R0 resection rates and disease-free survival, showed equivalence where reported.

Extended Colectomy vs. Segmental Resection for Transverse Colon Cancer

Mortality and morbidity: Perioperative mortality was consistently low (<1-2%) and comparable between robotic, laparoscopic, and open procedures [8,9]. Overall morbidity rates ranged from 11% to 20%, with no significant differences between surgical approaches [8,10]. Although robotic surgery sometimes had longer operative times, complication rates remained similar.

Anastomotic leak and ileus: Anastomotic leak rates were low and comparable across robotic and laparoscopic cohorts [8,9]. Postoperative ileus was rarely reported.

Oncologic outcomes: R0 resection rates were uniformly high (100%) for both robotic and laparoscopic surgery, indicating equivalent oncologic radicality [8,9]. Short-term disease-free and overall survival were comparable, though long-term survival data were limited.

Length of stay: Minimally invasive approaches (robotic and laparoscopic) were associated with shorter length of stay compared to open surgery [8,9]. Differences between robotic and laparoscopic groups were not statistically significant.

Extended Colectomy vs. Segmental Resection for Splenic Flexure Cancer

Mortality and morbidity: Mortality was very low (<1%) across segmental resections and left hemicolectomies, with no significant differences between robotic and laparoscopic surgery [11,12]. Overall morbidity ranged from 8% to 12%, with major complications similarly distributed [11,13].

Anastomotic leak and ileus: Anastomotic leaks were rare or absent [12]. Ileus occurred in 10-15% of patients, without significant differences between robotic and laparoscopic groups [12].

Reoperation and wound infection: Reoperation rates were low (3-5%) and comparable [11,14]. Wound infection rates were similarly low across approaches [12,13].

Length of stay: The median hospital stay was five to six days for both approaches, confirming comparable recovery times [11,12].

Oncologic outcomes: Two-year disease-free survival and short-term overall survival were equivalent for robotic and laparoscopic procedures [12]. R0 resection data were not consistently reported.

Methodological Quality and Bias Profile of the Included Studies

The risk of bias in all seven studies was evaluated using the ROBINS-I (Risk of Bias in Non-randomized Studies of Interventions) tool [15]. The seven studies demonstrated overall sound methodology. However, two primary limitations were identified using the ROBINS-I tool: a "moderate risk" of bias was consistently found in both the area of confounding variables (Domain 1) and in the selection of study participants (Domain 2). This indicates potential weaknesses in adequately controlling for external factors such as age, comorbidities, or disease severity, which might skew the findings.

For the remaining domains 3 through 7, the studies largely received "low risk" ratings, indicating robustness in areas like intervention classification and adherence, handling of missing data, outcome measurement, and results reporting. Table 2 summarizes the risk-of-bias assessment of the included studies.

**Table 2:** Risk-of-bias assessment using the ROBINS-I (Risk of Bias in Non-randomized Studies of Interventions) tool.

Study	Domain I (Confounding)	Domain 2 (Selection of Participants )	Domain 3 (Classification of Interventions)	Domain 4 (Deviations From Intended Interventions)	Domain 5 (Missing Data)	Domain 6 (Measurement of Outcomes)	Domain 7 (Selection of the Reported Result)
de’Angelis et al. [8]	Moderate	Moderate	Low	Low	Low	Low	Low
Milone et al. [9]	Moderate	Moderate	Low	Low to moderate	Low to moderate	Low	Low
Maertens et al. [10]	Moderate	Moderate	Low	Low to moderate	Low to moderate	Low	Low
Pang et al. [11]	Moderate	Moderate	Low	Low	Low	Low	Low
Zhang et al. [13]	Moderate	Moderate	Low	Low	Low	Low	Low
Kohn et al. [14]	Moderate	Moderate	Low	Low	Low	Low	Low
Kim et al. [12]	High	Moderate to high	Moderate	Low	Low	High	Low

Discussion

Principal Findings

This review demonstrates that robotic and laparoscopic colectomy yield broadly comparable outcomes for transverse colon and splenic flexure cancers. Mortality is uniformly low (<1%) across studies and datasets, with no significant differences between approaches [8-12,16]. Morbidity, including surgical site infection, anastomotic leak, postoperative ileus, and reoperation, remains statistically similar, with minor variations reported in population-based datasets not translating into meaningful clinical differences [8-10,15].

Oncologic adequacy is also equivalent. R0 resection rates are consistently high (>90-100%), and long-term overall and disease-free survival show no significant differences between robotic and laparoscopic procedures [8,9,16]. Robotic surgery may provide a modest reduction in hospital stay, likely due to enhanced precision and facilitation of intracorporeal anastomosis, though this is offset by longer operative times and higher costs [9-11,16].

For splenic flexure cancers, both segmental colectomy and extended left hemicolectomy demonstrate low morbidity and mortality with equivalent oncologic outcomes, supporting surgical equipoise [11,16]. Preoperative indocyanine green (ICG) fluorescence marking enhances surgical precision by facilitating intraoperative tumor localization and lymphatic mapping. When injected endoscopically into the submucosa, ICG remains visible under infrared light for up to a week, with detection rates around 97% [17]. The technique is safe, without major complications, and is increasingly used in both robotic and laparoscopic colorectal surgery [17].

Interpretation

Collectively, the evidence supports that minimally invasive colectomy, whether robotic or laparoscopic, is safe, effective, and oncologically adequate. Robotic approaches may offer minor perioperative advantages, particularly in recovery time and technical ease for complex dissections, but do not confer significant differences in survival or major complication rates. These findings are consistent across diverse clinical settings, including high-volume centers and population-based datasets, reinforcing the reproducibility of minimally invasive colorectal surgery worldwide.

Limitations

The majority of the included studies were observational and retrospective, increasing susceptibility to selection bias and unmeasured confounding. Small robotic sample sizes reduce statistical power, and heterogeneity in patient populations, surgical techniques, and outcome definitions limits comparability. Key outcomes, such as R0 resection rates for splenic flexure cancers, anastomotic leaks, wound infections, and long-term survival, were inconsistently reported. These factors preclude formal meta-analysis and may introduce publication bias. Additionally, the absence of prospective protocol registration may introduce a risk of selective reporting, although all outcomes were predefined prior to data extraction.

## Conclusions

In summary, robotic and laparoscopic colectomies yield comparable perioperative safety, complication rates, oncologic clearance, and survival. Robotics may provide modest advantages in terms of shorter hospital stays and technical maneuverability, but at the expense of increased operative time and cost. The decision between platforms should hinge on surgeon expertise, institutional resources, and patient-specific factors rather than perioperative risk. Future randomized controlled trials with mature long-term endpoints and cost-effectiveness analyses are needed to determine whether the recovery advantages of robotics justify its higher resource utilization, particularly in anatomically complex cases such as the transverse and splenic flexures of the colon.
